# Carbon Nanodots with Nearly Unity Fluorescent Efficiency Realized via Localized Excitons

**DOI:** 10.1002/advs.202302440

**Published:** 2023-07-26

**Authors:** Qing Lou, Qingchao Ni, Chunyao Niu, Jianyong Wei, Zhuangfei Zhang, Weixia Shen, Chenglong Shen, Chaochao Qin, Guangsong Zheng, Kaikai Liu, Jinhao Zang, Lin Dong, Chong‐Xin Shan

*Adv. Sci*. **2022**, *9*, 2203622

DOI: 10.1002/advs.202203622

**Figure 5:**
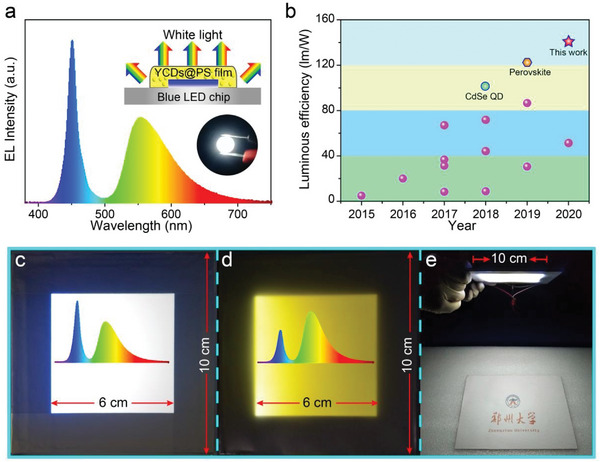
CD‐based WLEDs and flat‐panel illumination system. a) EL spectrum of the CD‐based WLED with a schematic diagram and photo of the WLED in the inset. b) Comparison of the LE of WLEDs based on CDs (purple points) and other materials. A detailed data point is presented in Table S5 (Supporting Information). c,d) Photograph of CD‐based area lights with c) cold and d) warm‐white light. The insets of panels (c) and (d) are the corresponding EL spectra. e) Picture under the flat‐panel illumination system with a light emitting area of 10 cm × 10 cm.

In the original published article there is a wrong point between 2015 and 2016 in the statistical diagram of Figure 5b. Please find below the correct **Figure** **5**.

